# Replacing BPA: Structural Substitutes BPAF Binding to the Progesterone Receptor Elevates Breast Cancer Risk

**DOI:** 10.1002/advs.202502444

**Published:** 2025-11-07

**Authors:** Xiaotong Ji, Peilin Li, Haoyang Wu, Linzhuo Shen, Xiaoyun Wu, Peiyun Jiang, Yating Li, Xiaozheng Zhang, Huifeng Yue

**Affiliations:** ^1^ Department of Environmental Health School of Public Health Shanxi Key Laboratory of Environmental Health Impairment and Prevention MOE Key Laboratory of Coal Environmental Pathogenicity and Prevention Shanxi Medical University Taiyuan Shanxi 030001 P. R. China; ^2^ College of Environment and Resource Research Center of Environment and Health Shanxi Key Laboratory of Coal‐based Emerging Pollutant Identification and Risk Control Shanxi University Taiyuan Shanxi 030006 P. R. China; ^3^ Department of Biochemistry and Molecular Biology Shanxi Key Laboratory of Birth Defect and Cell Regeneration Shanxi Medical University Taiyuan Shanxi 030001 P. R. China

**Keywords:** binding interaction, bisphenol analogs, breast cancer risk, progesterone receptor

## Abstract

As the global production of bisphenol analogs (BPs) surges to replace regulated bisphenol A (BPA), their pervasive environmental presence and uncharacterized breast cancer risk raise critical public health concerns. Herein, the environmental risks of BPs are deciphered by linking their structural affinity for the progesterone receptor (PR), a master regulator of breast cancer, to oncogenic outcomes across experimental tiers. Molecular simulations reveal that environmental BPAF and BPB exhibited stronger binding to the PR‐ligand binding domain (LBD) than BPA. Chemical assays confirm persistent PR‐LBD structural changes after BPs exposure, mimicking endocrine disruption patterns. The cellular thermal shift assay also confirms the interaction between the PR and BPs. In vitro, BPAF and BPF boost PR expression at human‐relevant concentrations. In addition, BPAF elevates PR expression, and the enhanced migratory and invasive capacities are effectively suppressed by the PR inhibitor. Toxicological Prioritization Index‐based risk stratification, weighted by the binding affinity of BPs to the PR and cellular toxicity, ranks BPAF as the highest‐risk analog. Alarmingly, low‐dose BPAF exposure (30 µg kg^−1^) accelerates mammary tumor growth in mice, paralleling PR upregulation in tumor tissues. This study underscores that substituting BPA with structurally akin analogs merely shifts, rather than mitigates, environmental health risks.

## Graphical abstract

This study demonstrates that bisphenol analogues, particularly BPAF, bind to progesterone receptor, promoting breast cancer cell proliferation, migration, and mammary tumor growth, indicating that bisphenol A substitutes may pose equal or greater health risks.

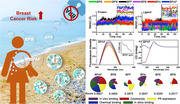

## 1 Introduction

Breast cancer is one of the most common cancers, and its incidence rate is the highest among malignant tumors in women. According to the latest global cancer burden data in 2020 released by the International Agency for Research on Cancer (IARC) of the World Health Organization, there were 2.26 million new cases of breast cancer worldwide by the end of 2020, exceeding lung cancer to become the world's largest number of new cancer cases. Hormones have been confirmed to be related to breast cancer, and 30% of these cases are responsive to therapies that involve altered hormones by ablation of the endocrine glands and the addition of hormone activators or inhibitors.^[^ ^1^ ^]^ Among these hormones, progesterone, with its proliferative effects, has been proposed to be closely related to the development of breast cancer via its respective receptors, the progesterone receptor (PR), which has been confirmed to regulate mammary growth and development.^[^ ^2^, ^3^ ^]^

Bisphenol A (BPA) is a well‐documented environmental endocrine‐disrupting chemical which has been demonstrated to be deleterious to human health. It is, therefore, being replaced by bisphenol analogs (BPs), including bisphenol AF (BPAF), bisphenol S (BPS), bisphenol B (BPB), bisphenol E (BPE), and bisphenol F (BPF). With the widespread use of these analogs, they are becoming increasingly ubiquitous in the environment.^[^ ^4^, ^5^ ^]^ In indoor dust, the concentration of total BPs was in the range of 0.026–111 µg g^−1^.^[^ ^6^ ^]^ This may be an important pathway for daily exposure accounting for 3.8%–35%.^[^ ^7^ ^]^ In food samples from the United States, the detection rates of BPA, BPS, BPF, and BPAF were 57%, 21%, 10%, and 11%, respectively.^[^ ^8^ ^]^ In addition, the estimated total daily intake of BPA and other BPs in China was in the range of 0.0048–0.75 and 0.646–0.664 µg kg^−1^ bw d^−1^, respectively.^[^ ^9^, ^10^ ^]^ The extensive environmental release and daily intake of these chemicals have led to a significant increase in the detection rate and concentration in human biological samples. Previous studies have indicated that BPs can be detected in human blood serum samples.^[^ ^9^, ^11^ ^]^ In addition, previous reports determined total BPs concentrations of 2.12 and 22.6 µg L^−1^ in urine samples from adults and children, respectively.^[^ ^12^, ^13^ ^]^

Due to the widespread detection of these substances in organisms, they have been associated with certain adverse effects on human health,^[^ ^14^, ^15^, ^16^ ^]^ the most important underlying mechanisms being their endocrine disrupting effects via types of hormone receptors including the steroid estrogen receptor (ERα, ERβ), androgen receptor (AR), thyroid hormone receptor (TR), peroxisome proliferator‐activated receptor gamma (PPARγ), and PR mentioned above.^[^ ^17^ ^]^ Exposure to low doses of BPA can lead to precocious neurogenesis and concomitant behavioral phenotype via androgen receptor‐mediated up‐regulation of aromatase.^[^ ^18^ ^]^ A study on the binding potential of bisphenol analogs with the ER showed that BPAF had 13 times higher estrogenic potency than BPA, while BPF and BPS showed approximately half of the potency^[^ ^19^ ^]^; other bisphenol analogs, such as BPS and BPF, also showed toxic and estrogenic effects similar to BPA.^[^ ^20^ ^]^ BPS and its analog, TBBPS, showed antagonistic activity to TRβ^[^ ^21^ ^]^; BPA and BPS altered the lipid metabolism by targeting PPARγ.^[^ ^22^ ^]^ Exposure to BPA, BPS, and BPF increases PR and suppresses spheroid attachment to Ishikawa cells.^[^ ^23^ ^]^ It is important to note that certain bisphenol analogs have been observed to demonstrate carcinogenic potency in the breast tissue by means of altering functional receptors. BPF and BPS induce protumorigenic changes in the morphology and proteome of human mammary gland organoids.^[^ ^24^ ^]^ ERα activation‐DNMT‐TET2‐DNA hydroxymethylation participates in stimulated proliferation of breast cancer cells induced by BPA or BPS.^[^ ^25^ ^]^ In addition to ER, PR is also a biomarker used routinely at diagnosis to characterize breast cancer, and participates in molecular subtyping and is a determining factor in treatment decisions. In breast cancer, PR should be considered as the specific receptor for bisphenol compounds. However, the role of the PR in breast cancer influenced by bisphenol analogs exposure has not been investigated, and the toxic potential of bisphenol analogs needs to be determined.

In this study, a novel research framework for PR‐related breast cancer influenced by exposure to bisphenol analogs was constructed using a systematic toxicological research strategy. We utilized published databases (Comparative Toxicogenomics Database (CTD) and Gene Expression Omnibus (GEO)), in silico calculations, chemical examinations, in vitro cellular assays, and Toxicological Prioritization Index (ToxPi) software to identify the molecular initiating events involved in bisphenol analogs exposure‐induced proliferation in human breast cancer cells, and ranked them based on environmental relative risk. Lastly, a nude mouse tumor model was used to further validate the pro‐breast cancer risk and PR expression of high‐risk bisphenol analogs.

## 2 Results

### 2.1 Biomarker Screening for Bisphenol‐Associated Breast Cancer

**Figure 1:**
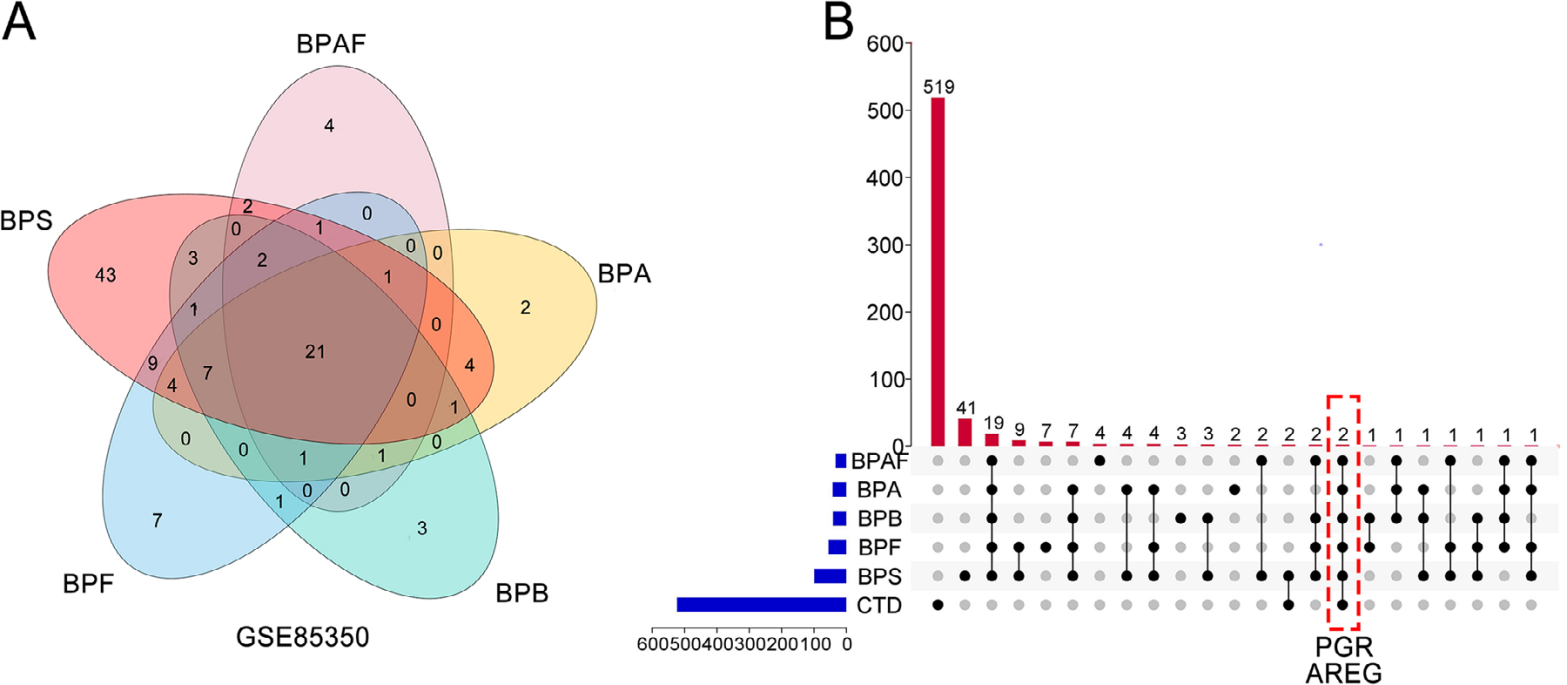
Biomarker screen for BPs‐associated breast cancer. A) The Venn diagram of the DEGs of the GSE85350 datasets. B) Upsets plots for DEGs shared by the GEO and CTD database. Blue bars in the left represent the numbers in different data sets. Red bars represent the shared numbers among different data sets. *PGR* is the gene name of PR.

We used a two‐stage screening approach to identify potential biomarkers of BPs‐associated breast carcinogenesis. Firstly, GSE85350 in the GEO database was subjected to re‐analysis. This high‐throughput screening study was based on a microarray of bisphenol analogs exposed to MCF‐7 cells. The objective of the re‐analysis was to screen for differentially expressed genes (DEGs) of BPA, BPB, BPE, BPF and BPAF, using the criteria of |log_2_FC| > 1 and *p* < 0.05 (**Figure** **1**A). We identify 523 breast cancer‐related genes associated with BPA exposure from the Comparative Toxicogenomics Database (CTD). Intersectional analysis of these two independent datasets revealed two consensus candidates: progesterone receptor (*PGR*) and amphiregulin (*AREG*) (Figure 1B). These overlapping genes represent potential molecular mediators, warranting further investigation into the interactions between BPs and breast cancer biomarkers.

### 2.2 In Silico Analysis of the Interaction between BPs and PR‐LBD

**Figure 2:**
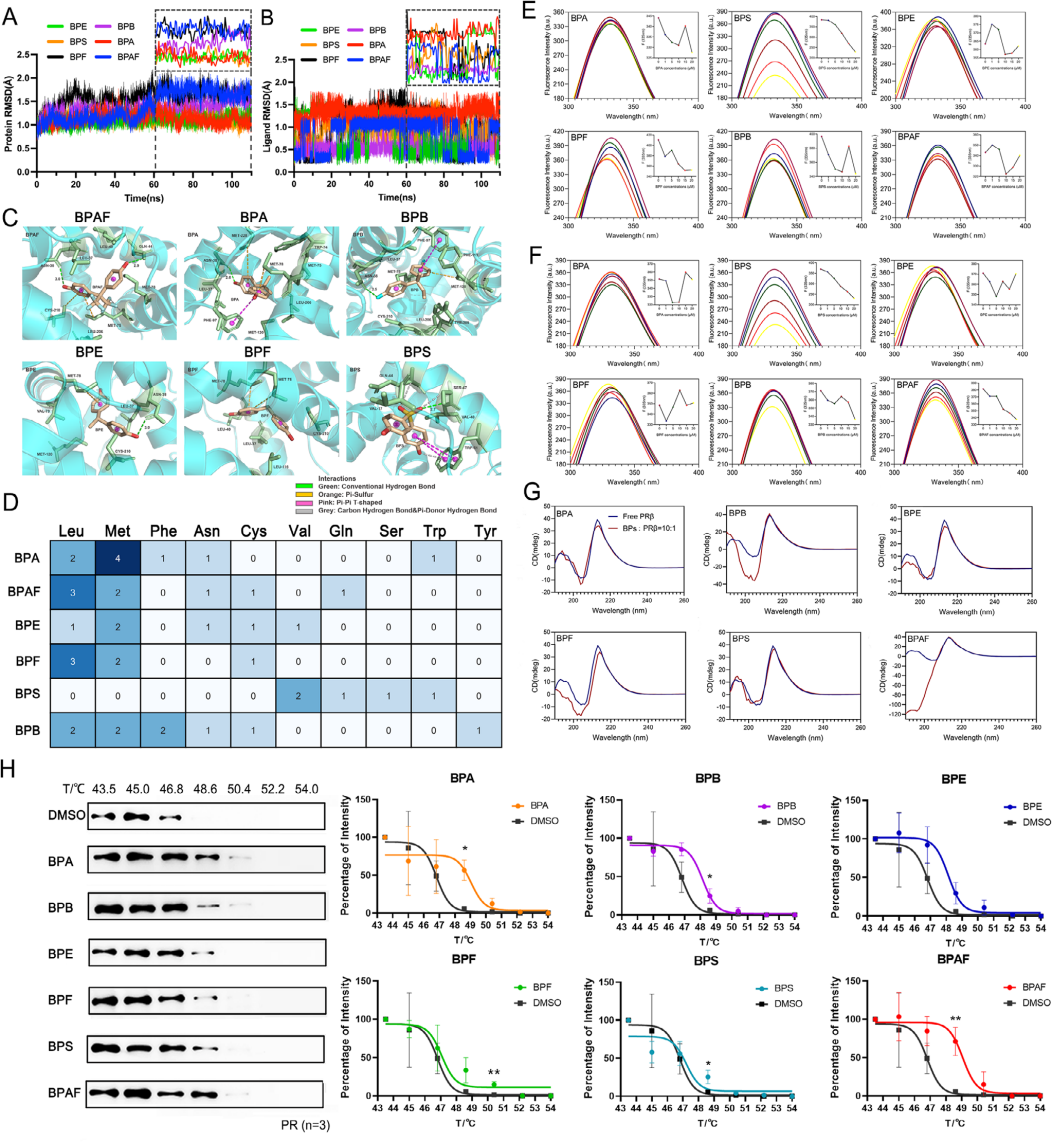
In silico, chemical, and in vitro analysis of the interaction between BPs and PR. Time evolution of Cα RMSD of A) PR‐LBD, and B) ligands during MD simulations. C) The binding mode of BPs with the PR‐LBD complex with lowest binding energy during MD simulations. D) Different hydrophobic amino acid residues surrounding bisphenol analogues within the PR‐LBD ligand‐binding cavity. Steady‐state fluorescence spectra of PR‐LBD in the presence of various concentrations of bisphenol analogs measured at E) 298K and F) 310 K; pH = 7.4; [PR‐LBD] = 0.5 × 10^−6^ m; [bisphenol analogs] = 0, 1, 5, 10, 15, 20 × 10^−6^ m. The excitation wavelength is set to 280 nm, and the emission wavelength is set to 300–400 nm. The excitation slit width was set to 5 nm, and the emission slit width was set to 10 nm. A broken line diagram shows the fluorescence intensity of bisphenol analogs at different concentrations at maximum emission wavelengths. G) CD spectra of PR‐LBD in the presence of bisphenol analogs measured at 298 K; pH = 7.4; [PR‐LBD] = 0.5 × 10^−6^ m; [bisphenol analogs] = 5 × 10^−6^ m. The scanning speed was set to 200 nm min^−1^, and the path length was set to 1 cm. The blue line represents the CD spectra of free TRs without ligand binding, and the red line represents the CD spectra of PR‐LBD after binding of bisphenol analogs. H) Blots from a representative experiment and the melting curves for PR treated with different BPs (*n* = 3). Data are presented as mean ± standard deviation (SD). *p < 0.05, **p < 0.01 versus Veh control. For CESTA data analysis, perform an unpaired two‐tailed *t*‐test using either Welch's correction (assumed unequal SDs) or the original unpaired two‐tailed *t*‐test (assumed equal population SDs).

First, the molecular docking results showed that BPAF exhibits the highest binding affinity (−8.6) toward PR, followed by BPB (−7.9), BPA (−7.8), BPE (−7.6), BPF (−7.6), and BPS (−7.6) (**Table** **1**). Furthermore, MD simulations were performed to validate the binding affinity of the bisphenol analogs to the PR. In MD simulations, the root mean square deviation of the α carbon atom (Cα RMSD) can reflect the stability of PR‐LBD binding with ligands.^[^ ^26^ ^]^ After 100 ns of MD simulations, BPA and its analogs to PR‐LBD systems reached relatively stable states (**Figure** **2**A,B). Overall, the combination of bisphenol analogs maintained initial RMSD values within 0.5–1.5 Å. PR‐LBD complexes with BPA, BPE, and BPS showed only minor fluctuations around 1.0 Å, indicating localized structural variations while maintaining global stability. In contrast, other bisphenol analogs induced more significant conformational changes in PR‐LBD, particularly BPAF, which displayed a notable Cα RMSD increase during 50–60 ns.

**Table 1:** Docking score of bisphenol analogs binding to PR‐LBD.

Chemicals	Docking score
BPA	−7.8
BPS	−7.6
BPE	−7.6
BPF	−7.6
BPB	−7.9
BPAF	−8.6

As shown in Figure 2C,D, key amino acid residues involved in PR‐LBD interactions differed among bisphenol analogs. Non‐specific hydrophobic amino acid residues, hydrogen bonds, and π‐sulfur interactions were the main factors of their interaction. Except for BPS and BPF, all ligands formed hydrogen bonds with Asn38, among which BPB showed the shortest hydrogen bond distance, while BPAF displayed the most conventional hydrogen bonds. Hydrophobic interactions with Leu37 were observed for all analogs except BPS. BPA and BPB were surrounded by the highest number of hydrophobic residues, followed by BPAF with eight residues (Gln44, Leu40, Met78, Met75, Leu37, Cys210, Asn38, Leu206). π‐Sulfur interactions were formed between PR‐LBD and all bisphenol analogs except BPS. Notably, BPS interacted with the fewest residues (Gln44, Val48, Trp51, Ser47, Val17).

The binding affinities of bisphenol analogs to the PR‐LBD were quantitatively described by calculating the binding free energy based on MM/GBSA (**Table** **2**). There was a negative correlation between the binding free energy and binding affinity, that is, the smaller the binding free energy means the stronger the binding affinity.^[^ ^27^ ^]^ The binding free energy of bisphenol analogs to the PR‐LBD was mainly a consequence of van der Waals interaction (Δ*E*_vdW_), electrostatic interactions (Δ*E*_ele_), polar solvation energy (Δ*G*_sol‐polar_), and nonpolar solvation energy (Δ*G*_sol‐nonpolar_), with Δ*E*_vdW_ being the main driving force of the combination. The order of binding affinity of bisphenol analogs to the PR‐LBD was BPAF > BPB > BPA > BPF > BPE >BPS. These results indicate that bisphenol analogs can interfere with the binding of natural ligands to the PR to varying degrees. Compared to other bisphenol analogs, BPAF had the lowest binding energies to the PR‐LBD, suggesting that stronger binding might induce the greater interference potential in the PR signaling pathway. These results above indicated that bisphenol analogs can stably bind to the PR‐LBD, and that more attention should be paid to their mechanisms of action.

**Table 2:** MM/GBSA‐derived binding free energies (kcal/mol) for bisphenol analogs binding to PR‐LBD.

Chemicals	Δ*E_vdW_*	Δ*E_ele_*	Δ*G_sol‐polar_*	Δ*G_sol‐nonpolar_*	Δ*G_binding_*
BPA	−33.24 ± 0.25	−15.18 ± 0.32	23.50 ± 0.20	−3.40 ± 0.01	−28.32 ± 0.27
BPAF	−36.91 ± 0.27	−14.92 ± 0.26	26.61 ± 0.18	−4.16 ± 0.01	−29.38 ± 0.24
BPE	−29.80 ± 0.22	−18.47 ± 0.40	26.43 ± 0.28	−3.28 ± 0.01	−25.11 ± 0.25
BPB	−35.50 ± 0.29	−13.42 ± 0.38	23.29 ± 0.29	−3.64 ± 0.01	−29.27 ± 0.27
BPS	−34.37 ± 0.23	−24.10 ± 0.56	40.04 ± 0.47	−3.33 ± 0.01	−21.76 ± 0.27
BPF	−32.40 ± 0.27	−17.49 ± 0.25	26.29 ± 0.17	−3.39 ± 0.01	−26.98 ± 0.28

Note: Numbers after ± present standard errors of mean.

### 2.3 BPs and PR‐LBD Interactions Based on FS Examination

Molecular docking and MD simulations results indicated possible binding modes between the bisphenol analogs and the PR; however, the binding mechanisms of the bisphenol analogs to the PR‐LBD still need to be explored. The steady‐state fluorescence spectra of the PR‐LBD without treatment with the bisphenol analogs showed a significant fluorescence peak at 335 nm for PR‐LBD (Figure 2E,F), which was consistent with the maximum emission center of tryptophan (Trp).^[^ ^28^ ^]^ The maximum emission wavelength of BPF shifted from 335 to 326 nm, whereas those of the other tested bisphenol analogs remained the same after titration. The maximum fluorescence emission wavelength did not show a significant red or blue shift, indicating that the Trp residue is exposed to a more hydrophilic environment or the shielding effect of the pollutant on the fluorophore; that is, after the titration of the pollutant, although it affects the microenvironment of the fluorophore, it does not change the region near the Trp residue.^[^ ^29^ ^]^

Simultaneously, we also observed a dose‐response relationship between fluorescence intensity and concentration of bisphenol analogs. For the PR‐LBD (Figure 2E,F), only the fluorescence intensity activated by BPS decreased with increasing concentration of BPs. The values of BPF at 298 K and of BPAF at 310 K were similar to those of BPS. Other bisphenol analogs had no dose‐effect relationship under any condition.

The molecular contact between fluorescent chromophores and quenchers includes static quenching, which is characterized by the formation of complexes, and dynamic quenching, which is characterized by effective collisions.^[^ ^30^ ^]^ Based on the above results, to further confirm the quenching mechanism induced by bisphenol analogs, the fluorescence quenching constant *k*_sv_ and the bimolecular quenching rate constant *k*_q_ at different temperatures (298 and 310 K) were calculated using the Stern–Volmer equation when fluorescence quenching occurred. In this study, the *k*_sv_ of PR‐LDB‐BPs systems titrated with BPA, BPS, and BPB decreased with increasing temperature (**Table** **3**), indicating that the stability of the complex formed by the bisphenol analogs and PR‐LBD decreased with increasing temperature, resulting in a decrease in the quenching constant. In addition, the *k*_q_ values were much larger than the maximum scattering collision quenching constants between the quenching agent and the biopolymer in the dynamic quenching (2 × 10^10^ L mol^−1^ s^−1^),^[^ ^31^ ^]^ again showing that the mechanism of protein quenching caused by bisphenol analogs was static quenching.

**Table 3:** Quenching constants, binding constants, and thermodynamic parameters of bisphenol analogs and PR‐LBD composites at different temperatures.

	Sample	*T* [K]	*K*_sv_×10^4^ [L mol^−1^]	*K*_q_×10^12^ [L mol^−1^ s^−1^]	*K*_a_×10^4^ [L mol^−1^]	Δ*H* [kJ mol^−1^]	Δ*G* [kJ mol^−1^]	Δ*S* [kJ mol^−1^ K^−1^]
PR‐LBD	BPA	298	0.76	0.76	0.98	84.510	−22.769	0.360
		310	0.66	0.66	3.67		−27.089	
	BPS	298	3.57	3.57	0.54	115.854	−21.293	0.460
		310	2.94	2.94	3.30		−26.815	
	BPE	298	‐	‐	‐	‐	‐	‐
		310	1.06	1.06	2.30		−25.885	
	BPF	298	0.51	0.51	1.41	‐	−23.671	‐
		310	‐	‐	‐		‐	
	BPB	298	1.01	1.01	10.51	−82.412	−28.647	−0.180
		310	0.38	0.38	2.90		−26.482	
	BPAF	298	‐	‐	4.16	−25.568	−26.351	0.003
		310	0.58	0.58	2.79		−26.383	

The PR is a ligand‐dependent transcription factor,^[^ ^32^ ^]^ and the binding affinity between the ligand and proteins will affect the downstream transcriptional activity of proteins.^[^ ^33^ ^]^ As indicated in Table 3, the binding constants k_a_ of bisphenol analogs and PR‐LBDs were in the optimal range of 10^4^–10^6^ L mol^−1^, indicating that bisphenol analogs bind the PR more easily and interfere with the action of endogenous natural ligands.^[^ ^4^ ^]^ The order of PR‐LBD binding affinity at 298 K (no value for BPE) was BPB > BPAF > BPF > BPA > BPS; for that at 310 K, the order of binding affinity was BPA > BPS > BPB > BPAF > BPE. Compared with other bisphenol analogs, BPB and BPAF has a stronger binding constant with the PR‐LBD. Such tight binding may affect the physiological function of the protein, result in stronger toxicity. According to the thermodynamic parameters, the complexation properties between the ligand and receptor can be roughly determined, generally including van der Waals forces, hydrogen bonds, hydrophobic interactions, and electrostatic interactions.^[^ ^29^ ^]^ The change in free energy of the composite system, Δ*G* < 0, indicates that the binding of bisphenol analogs to the PR‐LBD was a spontaneous process. Van der Waals forces and hydrogen bonds played major roles in the binding of the PR‐LBD to BPB (Δ*H* < 0 and Δ*S* < 0), while the binding of BPA and BPS to the PR‐LBD was mainly driven by hydrophobic interactions (Δ*H* > 0 and Δ*S* > 0).

### 2.4 CD Spectra in Determining the Interaction between BPs and the PR‐LBD

Changes in the fluorescence characteristics of the PR‐LBD caused by the addition of bisphenol analogs have been reported. However, it is unclear whether this binding leads to conformational changes in the proteins. Therefore, CD was used to estimate the secondary structure of the proteins.^[^ ^34^ ^]^ As shown in Figure 2G and **Table** **4**, the addition of bisphenol analogs decreased the band strength of the PR‐LBD and all the PR‐LBD‐BPs, composite systems, except BPB, showed a decrease in α‐helix. The titration of BPAF caused the most intense conformational change in random coil (20.35% reduction) and β‐sheet (20.83% increase). The BPB and BPF values also significantly changed. This could be attributed to the disruption of the intramolecular forces of the PR‐LBD by the electrostatic interactions of some amino acid residues induced by bisphenol analogs and hydrogen bonding between peptides, leading to the transformation of the helical structure to a more extended sheet structure.^[^ ^35^ ^]^ In the binding study of BPAF and human serum albumin (HAS), BPAF reduced the α‐helix content of HAS by destroying the hydrogen bond network.^[^ ^36^ ^]^

**Table 4:** Secondary structure content of PR‐LBD in the presence of bisphenol analogs.

Sample	c(BPs) / c(PR‐LBD)	Secondary structural elements in PR‐LBD			
		α‐helix (%)	β‐sheet (%)	β‐turn (%)	Random coil (%)
BPA	0:1	9.39	44.64	14.48	31.55
	10:1	9.15	44.24	14.22	32.39
BPS	10:1	8.91	46.03	14.37	30.69
BPE	10:1	7.88	48.59	14.13	29.39
BPF	10:1	8.38	52.89	15.11	23.62
BPB	10:1	10.60	55.50	16.75	17.15
BPAF	10:1	2.40	65.47	20.93	11.20

### 2.5 Effects of BPs Exposure on the Thermal Stability of the PR in MCF‐7 Cells

Using CETSA, we demonstrated that bisphenol analogs enhanced the thermal stability of the PR protein in MCF‐7 cells. Preliminary temperature gradient experiments revealed a progressive decrease in soluble PR levels with increasing temperature, whereas all bisphenol analogs compounds demonstrated rightward shifts in the thermal denaturation curves (Figure 2H **)**. The thermal transition midpoint (Tm50), defined as the temperature required to precipitate 50% of total PR protein, showed a positive shift in BPs‐exposed groups compared to controls, with differential values calculated as ΔTm50 (Table S1, Supporting Information). BPA induced the most substantial thermal stabilization effect with a ΔTm50 = 1.97 °C, followed by BPAF (ΔTm50 = 1.89 °C), while BPS and BPF showed comparatively moderate stabilization. Collectively, these data provide consistent evidence that bisphenol derivatives enhance the PR thermal stability in a compound‐specific manner.

### 2.6 Cytotoxicity and PR Expression Levels Induced by BPs

**Figure 3:**
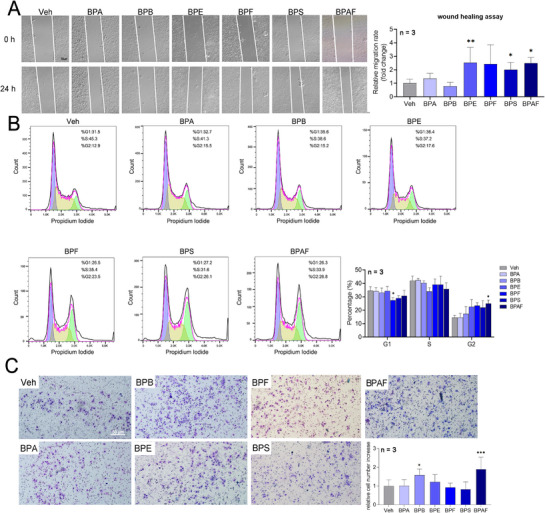
The effects of bisphenol analogs on the cytotoxicity of MCF‐7 cells. A) Representative images and statistical results of wound healing assay of bisphenol analogs on MCF‐7 cells at 10^−10^ m (*n* = 3). One‐way ANOVA with Dunnett's correction for multiple comparisons was used, assuming equal SDs. B) Representative images and statistical results of cell cycle assay of bisphenol analogs on MCF‐7 cells at 10^−10^ m (*n* = 3). One‐way ANOVA with Dunnett's correction for multiple comparisons was used, assuming equal SDs. C) Representative images and statistical results of the transwell assay of bisphenol analogs on MCF‐7 cells at 10^−10^ m (*n* = 3). One‐way ANOVA with Dunnett's correction for multiple comparisons was used, assuming equal SDs. Data are presented as mean ± standard deviation (SD). *p < 0.05, **p < 0.01, ***p < 0.001 versus Veh control.

The CCK‐8 results (Figure S1, Supporting Information) showed that all BPs promoted MCF‐7 cell proliferation at concentrations as low as 10^−10^ m, which was identified as the minimum effective dose. Given the critical role of cell migration in tumor metastasis, we evaluated BPs‐induced motility using a wound healing assay (**Figure** **3**A). After 24 h of treatment, the relative migration rate increased to 2.54 (BPE), 2.00 (BPS), and 2.50 (BPAF) of the control, indicating enhanced migratory capacity across all BPs‐exposed groups. To investigate cell cycle dynamics, flow cytometry analysis demonstrated that BPF significantly reduced the proportion of G1‐phase cells, while BPAF increased the G2/M‐phase population (Figure 3B). These findings suggest that BPs accelerate MCF‐7 cell cycle progression. Furthermore, transwell invasion assays revealed that BPAF exposure increased the invasive capacity by 2.06‐fold compared to that of the control (Figure 3C).

**Figure 4:**
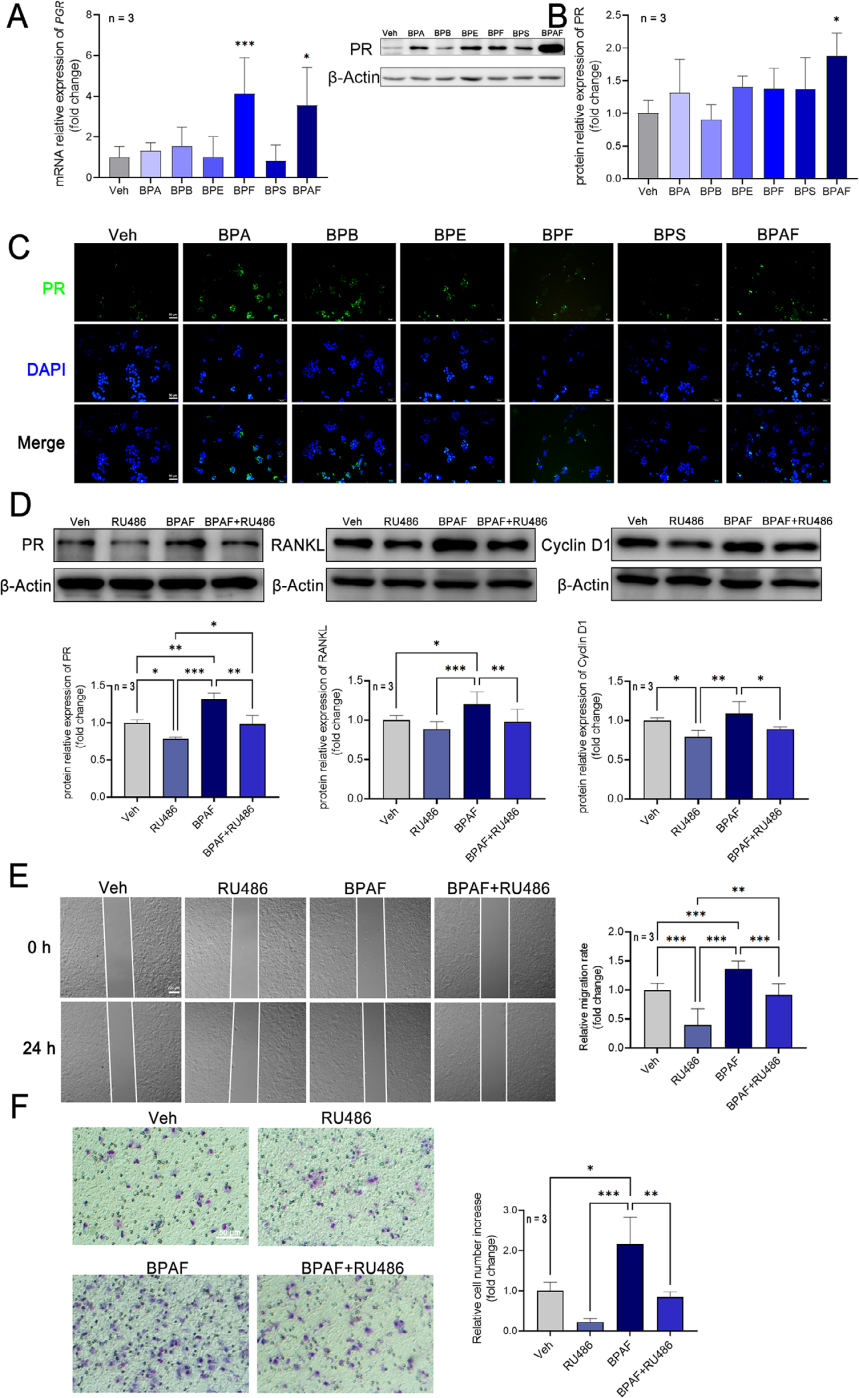
The effects of bisphenol analogs on Progesterone Receptor expression. A) The effects of bisphenol analogs on *PGR* mRNA expression of MCF‐7 cells at 10^−10^ m (*n* = 3). One‐way ANOVA with Dunnett's correction for multiple comparisons was used, assuming equal SDs. B) The effects of bisphenol analogs on PR protein expression of MCF‐7 cells at 10^−10^ m (*n* = 3). One‐way ANOVA with Dunnett's correction for multiple comparisons was used, assuming equal SDs. C) Representative images of IF staining of PR of MCF‐7 cells treated with different bisphenol analogs at 10^−10^ m (*n* = 3). D) The effects of BPAF and RU486 on PR, RNAKL, and Cyclin D1 protein expression of MCF‐7 cells at 10^−10^ m (*n* = 3). One‐way ANOVA with Dunnett's correction for multiple comparisons was used, assuming equal SDs. E) Representative images and statistical results of wound healing assay of BPAF and RU486 on MCF‐7 cells (*n* = 3). Brown–Forsythe and Welch ANOVA tests with Dunnett's T3 correction were used, assuming unequal SDs. F) Representative images and statistical results of the transwell assay of BPAF and RU486 on MCF‐7 cells (*n* = 3). Brown–Forsythe and Welch ANOVA tests with Dunnett's T3 correction were used, assuming unequal SDs. Data are presented as mean ± SD. *p < 0.05, **p < 0.01, ***p < 0.001 versus corresponding group.

Subsequent analysis of PR expression revealed differential regulation by BPs. Quantitative RT‐PCR showed that BPF and BPAF significantly upregulated *PGR* mRNA levels by 4.12‐fold and 3.55‐fold, respectively (**Figure** **4**A). However, western blot analysis showed that only BPAF induced a 1.88‐fold increase in PR protein expression (Figure 4B). Immunofluorescence staining further confirmed elevated PR expression in BPAF‐treated cells compared to that in the controls (Figure 4C). Collectively, these data demonstrated that BPs enhance MCF‐7 cell proliferation, migration, and invasion, while modulating cell cycle progression and PR expression.

Furthermore, treatment with the PR inhibitor RU486 abrogated the BPAF‐induced upregulation of PR expression and its downstream transcriptional targets, RANKL and Cyclin D1 (Figure 4D). In addition, the enhanced migratory and invasive capacities resulting from BPAF exposure were effectively suppressed following RU486 administration (Figure 4E,F).

### 2.7 Systematic Evaluation of the Risk of BPs

**Figure 5:**
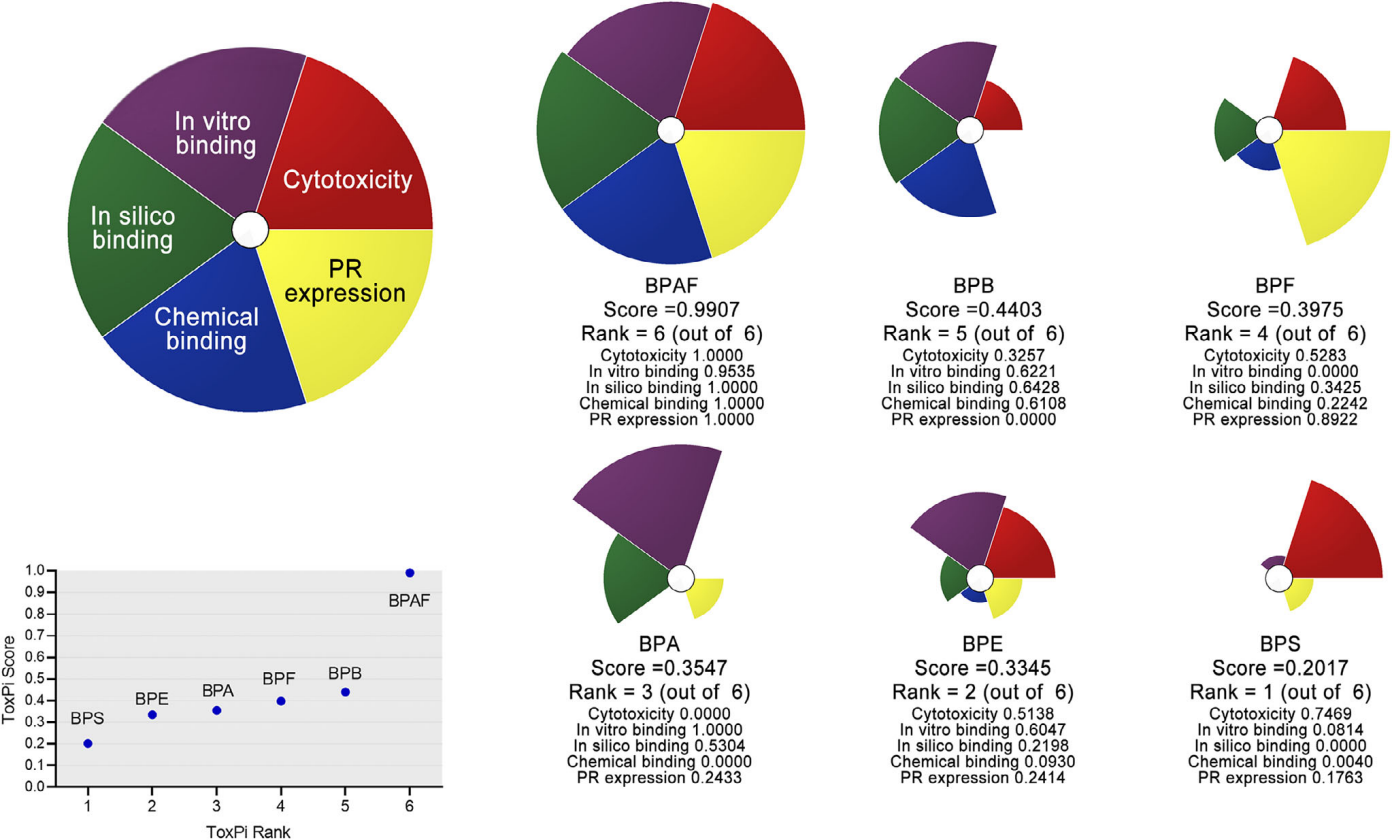
Ranking the toxic potential of bisphenol analogs by systematic evaluation using ToxPi.

We conducted a comprehensive risk analysis using ToxPi software for these chemical substances. As shown in **Figure** **5**, BPAF poses the highest risk, followed by BPB, BPF, BPA, BPE, and BPS. The main risk factors for BPAF include PR expression, cytotoxicity, and in silico and in vitro binding affinity, whereas for BPB, the primary risk factors are in silico binding, in vitro binding, and chemical binding. The risk factors for BPF and BPA are PR expression and in vitro binding, respectively.

### 2.8 In Vivo Assessment of BPAF Tumor‐Promoting Potential in a Xenograft Model

**Figure 6:**
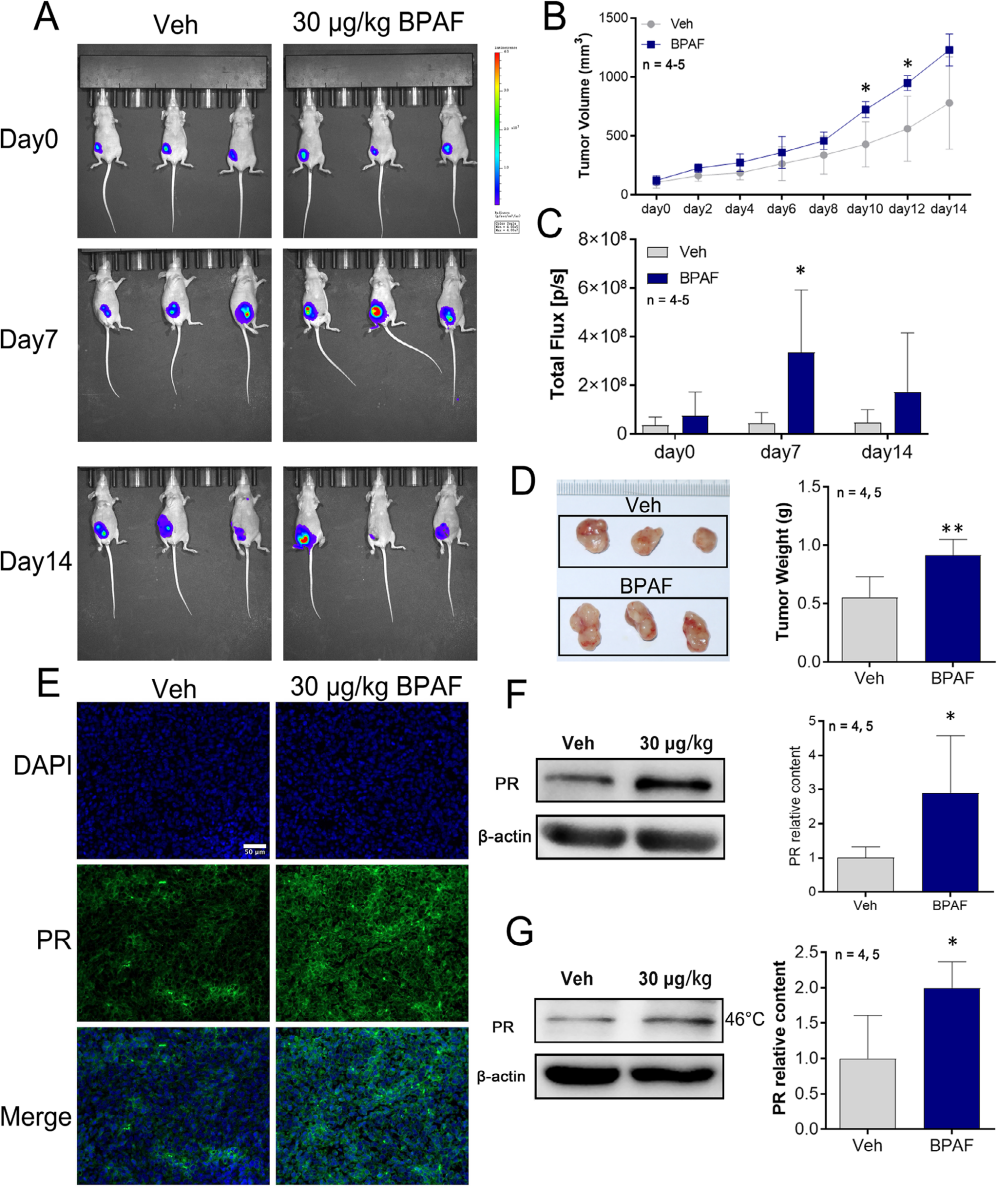
Protumor efficacy and analysis of PR protein expression and thermal stability in nude mice xenograft models. A) in vivo bioluminescence imaging of tumor‐bearing nude mice at the indicated time points (*n* = 4–5/group). B) Tumor growth curves measured by volume (*n* = 4–5/group). The data of day 0, 2, 4, 6, 8, 10, and 14 were analyzed using an unpaired two‐tailed *t*‐test, assuming both populations have the same SDs. The data from day 12 were analyzed using an unpaired two‐tailed *t*‐test with Welch's correction, assuming both populations have unequal variances. C) Total flux quantification from bioluminescence signals (*n* = 4–5/group). An unpaired two‐tailed *t*‐test with Welch's correction was used, assuming populations have unequal SDs. D) Excised tumor specimens (left panel) and corresponding tumor weights (*n* = 4–5/group). Unpaired two‐tailed *t*‐test, assuming populations have equal SD. E) Immunofluorescence staining of PR protein (green) in tumor sections; nuclei counterstained with DAPI (blue) (*n* = 3). Scale bar: 50 µm. F) Western blot analysis of PR expression levels. β‐actin served as a loading control (*n* = 4–5/group). An unpaired two‐tailed *t*‐test was used, assuming populations have equal SD. G) Thermal shift assay showing PR protein stability under 46 °C (*n* = 4–5/group). An unpaired two‐tailed *t*‐test was used, assuming populations have equal SD. Data are presented as mean ± SD. *p < 0.05, **p < 0.01 versus Veh control.

To investigate the oncogenic effects of bisphenol analogs in vivo, female nude mice were subcutaneously inoculated with PR‐overexpressing MCF‐7 cells and administered BPAF (30 µg kg^−1^ d^−1^) which rank the first of these six bisphenol analogs for 2 weeks. Tumor progression was quantified using bioluminescence imaging and caliper measurements. BPAF exposure did not change the body weight of the mice during the exposure periods (Figure S2, Supporting Information). However, BPAF‐exposed mice exhibited accelerated tumor growth kinetics, with average tumor volumes reaching 1230 mm^3^ by day 14 compared to 781 mm^3^ in controls (**Figure** **6**A,B). Bioluminescence imaging further revealed a 7.5‐fold increase in total photon flux (3.36 × 10^8^ photons s^−1^ vs 4.46 × 10^7^ photons s^−1^) in the BPAF group relative to controls on day 7 (Figure 6C). At the experimental endpoint, BPAF‐treated tumors demonstrated a 1.64‐fold increase in weight compared to the controls (*p* < 0.01, Figure 6D). Immunofluorescence staining confirmed elevated PR expression in BPAF‐treated tumor tissues compared to that in controls (Figure 6E). In addition, the results shown in Figure 6F demonstrated that exposure to 30 µg kg^−1^ of BPAF leads to an increase in PR protein levels, reaching 2.89‐fold of the control group. Thermal shift assays (Figure 6G) corroborated these findings, showing stabilization of PR protein at 46 °C in BPAF‐exposed tumors, suggesting enhanced structural stability and ligand‐binding affinity. Collectively, these results demonstrate that BPAF exposure promotes tumor growth in vivo, concomitant with the PR overexpression and protein stabilization, highlighting PR as a potential mediator of BPAF‐driven oncogenicity.

## 3 Discussion

Global substitution of BPA with structurally analogous alternatives has inadvertently created a new frontier of environmental health hazards, as evidenced by our multilevel dissection of the PR‐dependent mammary carcinogenicity of bisphenol analogs. Our findings challenge the presumption of safety in chemical substitution by demonstrating that prevalent analogs, notably BPAF, exhibit enhanced PR‐binding potency compared to BPA, which directly correlates with oncogenic outcomes. Molecular dynamics (MD) simulations revealed that the superior binding of BPAF to the PR LBD induces structural disturbance (e.g., α‐helix‐to‐β‐sheet transitions), a hallmark of endocrine‐disrupting activity. Critically, CETSA confirmed sustained PR‐analog interactions in vitro, whereas ToxPi modeling integrating binding affinity and cytotoxicity metrics identified BPAF as the highest‐risk analog. Strikingly, even at a dose of 30 µg kg^−1^, BPAF exposure drove PR overexpression and accelerated mammary tumorigenesis in vivo. These data collectively reveal a pernicious cycle: the structural mimicry of bisphenol analogs enables PR hyperexpression, fueling breast carcinogenesis while evading regulatory scrutiny.

Risk factors for breast cancer include age, family history, inherited gene mutations (such as BRCA1 and BRCA2), hormonal factors (early puberty, late menopause, hormone replacement therapy), obesity, alcohol consumption, and exposure to endocrine‐disrupting chemicals such as BPA. Breast cancer is often assessed by evaluating activation or inhibition of receptors such as ERα and PR.^[^ ^37^, ^38^ ^]^ PR expression is estrogen‐dependent and can modulate the ER activity.^[^ ^39^ ^]^ Regarding the role of BPA in breast cancer risk, studies have mainly focused on the binding affinity and activity of bisphenol analogs in human nuclear ER,^[^ ^40^ ^]^ estrogen‐related receptor gamma,^[^ ^27^, ^41^ ^]^ GR, AR, and RXR.^[^ ^42^ ^]^ As early as the 1990s, the determination of PR mRNA and protein expression was primarily used to examine the effects of BPA. Subsequently, computational toxicology approaches, E‐screen assays, and in vitro bioassays have been increasingly utilized to assess these interactions, as well as their potential agonistic or antagonistic effects. In addition, microarray and sequencing technologies have been employed to screen for changes in the gene expression of cancer cells altered by bisphenol analogs. The potential links between PR and bisphenol analogs that underlie breast cancer development, as well as the risks associated with different bisphenol compounds, require further investigation.

The CTD and GEO databases provided a robust strategy for conducting large‐scale data processing and screening of genes related to breast cancer in association with bisphenol analogs, and the PR is considered a target gene that is related to breast cancer. As early as 1993, Krishnan et al. suggested that BPA (10–25 × 10^−9^ m) induces PRs in MCF‐7 cells.^[^ ^43^ ^]^ In addition, BPA alters the expression of the PR in other cells, such as endometrial carcinoma cell lines^[^ ^23^, ^44^ ^]^ and human uterine stromal fibroblast cells.^[^ ^45^ ^]^ in vivo, BPA‐exposed rat mammary glands show lower PR expression,^[^ ^46^ ^]^ but perinatal exposure to BPA increases PR expression in the mammary gland of 6‐month‐old Mongolian gerbils.^[^ ^47^ ^]^ A previous study also indicated that bisphenol analogs enhance PR expressions as indicated by a stable luciferase PR reporter transfected into MVLN cells.^[^ ^48^ ^]^ Research based on microarrays of MCF‐7 cells treated with bisphenol analogs indicated that BPA, BPAF, BPB, BPF, BPZ, BPS, and BPAP consistently increase PR mRNA expression.^[^ ^49^ ^]^ BPA‐induced PR was antagonized by ER antagonist, ICI 182780, suggesting an ER‐mediated pathway.^[^ ^44^ ^]^ Previous studies have also linked bisphenol analogs exposure with the up‐regulated expression of the PR in vitro; However, they mainly focused on their ER‐related estrogenicity.^[^ ^50^, ^51^ ^]^ Importantly, BPS exposure significantly increased MCF‐7 proliferation to more than 1.4‐fold of the vehicle control,^[^ ^52^ ^]^ and BPA and four of the bisphenol analogs, including BPAF, enhanced the proliferative potency of MCF‐7 cells with increased PR expression.^[^ ^51^ ^]^ However, another study pointed out that BPA was neither able to induce the proliferation of MCF‐7 nor affect PR mRNA expression.^[^ ^53^ ^]^ To confirm the role and chemical mechanism of the PR in breast cancer induced by bisphenol analogs, it is necessary to explore the possible interactions between bisphenol analogs and the PR, including binding affinity and conformational changes.

Our results show that bisphenol analogs stably bind to the PR by interacting with the amino acid residue of the receptor. Leu and Met are the key residues that accommodate the binding of exogenous bisphenol analogs to natural proteins, which have been reported in the studies on pollutants, thyroxine binding globulin (TBG), epidermal growth factor receptor, AR, and glucocorticoid receptor.^[^ ^54^, ^55^, ^56^ ^]^ Van der Waals interactions were also observed as the dominant mechanism in MD simulations of the binding characteristics of pentabromoethylbenzene and BPS analogs to thyroid hormone receptors (TRβ‐LBD).^[^ ^21^, ^57^ ^]^ Fluorescence spectroscopy (FS) and CD are characterizations for the in vitro detection of the binding potential and conformational changes between chemicals and receptor proteins. Our study and other previous studies^[^ ^21^, ^58^ ^]^ found that endocrine‐disrupting chemicals lead to enhanced fluorescence intensity with increased concentrations because they might have higher hydrophobic interactions. The hydrophobic binding cavity of the protein restricts its rotation, improves the fluorescence quantum yield, and then increases the fluorescence intensity when bound with protein‐LBDs. Moreover, the addition of bisphenol may cause the stretching of peptide chains in proteins, resulting in an increase in distance and a decrease in energy transfer efficiency.^[^ ^59^ ^]^ Endogenous fluorescence quenching of Trp residues in natural proteins is often used to monitor the mechanism of protein‐ligand binding, and studies have shown that the binding region of bisphenol is mainly located near Trp.^[^ ^59^ ^]^ It was reported that the selective sensitization of bovine serum albumin (BSA) was also observed in the combination of BSA and doxycycline (DC).^[^ ^60^ ^]^ ─OH and ─S═O on the two aromatic rings connect the hydrophobic benzene ring to increase the interaction between BPS and the protein amino acid residues, resulting in tighter binding,^[^ ^61^ ^]^ which was consistent with the significant fluorescence quenching in our results. These altered bindings further led to a changed secondary structure in the receptor protein. Similar to our finding, Ikhlas et al. also found that compared with the parent compound BPA, BPB induced stronger conformational changes and greater damage to BSA.^[^ ^59^ ^]^

Stable binding by BPs to PR may lead to the sustained transcriptional upregulation of genes such as RANKL, a well‐established key mediator of progesterone‐induced mammary epithelial cell proliferation and a known driver in breast cancer pathogenesis. In addition, it may modulate the expression of genes involved in cell‐cycle progression (Cyclin D1), which is a fundamental process underlying cell migration and invasion.^[^ ^39^ ^]^ Our previous studies found that bisphenol analogs exposure could lead to abnormal cell viability and/or proliferation, alter the conformation and receptor–ligand binding of bisphenol analogs and the typical proteins,^[^ ^58^, ^62^ ^]^ but the results of each single experiment are not completely consistent. In addition, an increasing number of databases have provided abundant data, including chemical information, exposure concentrations, and toxicity data. How can these data be integrated, and the risks of chemical compounds be ranked to provide a reference for the government in prioritizing pollutants? Recently, a study reviewed risk ranking methods, which can be classified as quantitative, semi‐quantitative, and qualitative.^[^ ^63^ ^]^ For chemical hazards, risk ratios, scoring, flowcharts, and risk matrices have been considered for ranking. Recently, the ToxPi framework has provided a graphical system for analyzing complex toxicological data through incorporating data from in vitro assays, chemical descriptors, and biological pathways. Based on the risk ranking analysis and ToxPi software, Zhao et al. integrated exposure level with toxicity data based on ToxCast database, and the results showed that BPA (8/7770), BPAF (12/7770), and BPB (14/7770) showed a higher priority ranking; the risk of BPE (682/7770), BPF (1041/7770), and BPS (1522/7770) was relatively lower.^[^ ^64^ ^]^ Recently, Gao et al. proposed an integrated chemical risk ranking score system for shortlisting chemicals of concern in relation to the risk of preeclampsia, and BPA, perfluorooctanoic acid, and 3‐phenoxybenzoic acid are the main risk factors. This mode of precision prioritization can be applied in developing other disease‐specific substance priority lists.^[^ ^65^ ^]^ In the present study, we prioritized bisphenol compounds for breast cancer by integrating computational modeling, chemical binding analysis, and in vitro validation, and found that the comprehensive toxicity of bisphenol analogs such as BPAF and BPB may be higher than that of BPA itself. Therefore, BPAF was chosen for in vivo experiments. The results showed that 30 µg/kg BPAF exposure also had a pro‐proliferative effect on MCF‐7 in vivo, and that there was a significant increase in the expression of PR and binding to PR. Reference on the promotion of PR‐positive cell proliferation by BPAF in vivo is relatively lacking, and only a reference on the promotion of ERα‐negative SKBR‐3 breast cancer cells proliferation by BPAF in vivo was found.^[^ ^66^ ^]^ As for BPA, maternal BPA exposure promotes MCF‐7 cells injected into the flanks of ovariectomized mice to form tumors by 7 weeks post‐transplantation.^[^ ^67^ ^]^ While our study provides compelling evidence for the oncogenic potential of BPAF through PR‐mediated pathways in vivo, it is important to acknowledge its limitation. The in vivo validation was conducted exclusively with BPAF, which was identified as the highest‐risk analog by our integrated ToxPi model. Consequently, the direct in vivo mammary oncogenic effects of other bisphenol analogs, such as BPB, which also demonstrated significant PR‐binding and cellular activities in our earlier experiments, remain to be fully characterized.

In conclusion, our findings demonstrate that bisphenol analogs, widely used as substitutes for BPA, exhibit stronger binding affinity to the PR and induce structural changes, mimicking endocrine disruption patterns. These bisphenol analogs, particularly BPAF, not only upregulate PR expression in vitro but also promote mammary tumor growth in vivo. These findings highlight a concerning environmental feedback loop wherein the pervasive presence of bisphenol analogs contributes to PR‐driven breast carcinogenesis, exacerbating public health risks. Our results emphasize that the replacement of BPA with structurally similar analogs does not mitigate, but rather shifts environmental health hazards.

## 4 Experimental Section

### Chemicals and Kits

BPA (purity > 99%) and other analogs, namely, BPS (purity > 98%), BPE (purity > 98%), BPF (purity > 99%), BPB (purity > 98%), and BPAF (purity > 98%) were purchased from Tokyo Chemical Industry Co., Ltd. (Japan). Stock solutions of BPs were prepared in dimethyl sulfoxide (DMSO) at 0.1 m. PR‐ligand binding domain (LBD) was synthesized by Zoonbio Biotechnology Co., Ltd. (Nanjing, China). MCF‐7, a breast cancer cell line, and its special culture medium for MCF‐7 (MEM containing NEAA, 10 µg mL^−1^ insulin, 10% FBS, and 1% P/S) were purchased from Procell Life Science & Technology Co., Ltd. (Wuhan, China). Anti‐Progesterone receptor rabbit pAb was purchased from Wuhan Servicebio Technology Co., Ltd. (Wuhan, China), and the fluorescent secondary antibody was purchased from Beijing Biosynthesis Biotechnology Co., Ltd. (Beijing, China).

### Molecular Docking and Molecular Dynamics Simulations

PR‐LBD X‐ray structure was obtained (PDB ID: 1A28) from the Protein Data Bank (PDB) (http://www.rcsb.org/pdb/). The missing hydrogen atoms were added at pH 7.0. Ligands (BPA, BPS, BPE, BPF, BPB, and BPAF) were constructed to perform the molecular docking by AutoDock Vina,^[^ ^68^ ^]^ in which the Iterated Local Search Globule Optimizer^[^ ^69^, ^70^ ^]^ was applied to locate the most favorable binding sites. Here, PR were treated as rigid bodies, semiflexible molecular docking was carried out, and optimal binding sites were searched. The top poses for each ligand were selected by using the scoring function in AutoDock Vina.

The best binding pose of each ligand with PR was further refined using MD simulations. MD simulations were conducted using Amber 24 PMEMD2024. The optimal docking complexes extracted from molecular docking were parameterized with the AMBER ff14SB force field (protein) and GAFF2 force field (ligands). Atomic charges of ligands were assigned via the AM1‐BCC method in the antechamber module, with missing force field parameters generated by parmchk2. Each system was solvated in a truncated octahedral TIP3P water box with a 12.0 Å buffer distance, neutralized with Na^+^/Cl^−^ ions. Energy minimization was performed in two stages: first, 1000 steps of optimization with 500 kcal mol^−1^ Å^−2^ harmonic restraints applied to protein backbone atoms, followed by 50 000 steps of minimization without constraints. Under *NVT* ensemble, the system was gradually heated from 0 to 310 K over 300 ps with 10 kcal mol^−1^ Å^−2^ restraints on backbone atoms, followed by 10 ns *NPT* equilibration at 1 atm. Finally, 100 ns production simulations were executed under *NPT* conditions (310 K, 1 atm) using a 2 fs timestep with SHAKE algorithm constraints on hydrogen bonds. Long‐range electrostatic interactions were treated using the PME method with a 10 Å cutoff.

The convergence of MD simulations for the complexes was evaluated by the root mean square deviation (RMSD) of backbone atoms after superposition.^[^ ^71^ ^]^ Based on the 100 snapshots extracted from the last 5 ns equilibrated MD trajectory, the binding free energies of each ligand in complex with PR were calculated using the Molecular Mechanics Generalized Born Surface Area (MM−GBSA) method. The complex conformation with minimal potential energy was extracted using CPPTRAJ for ligand–protein interaction analysis.

### Fluorescence Spectroscopy Examination

The PR‐LBD was combined with bisphenol analogs at different concentrations and incubated for 30 min at 298 or 310 K. The emission spectra of the PR‐LBD were recorded from 300 to 400 nm under 280 nm excitation using a spectrofluorometer (Agilent Cary Eclipse). The fluorescence value of the buffer solution was recorded to eliminate background noise. The binding fluorescence spectra were obtained by smoothing using GraphPad Prism, and the binding parameters were calculated as we described previously.^[^ ^58^ ^]^

The fluorescence quenching constant *k*_sv_ and the bimolecular quenching rate constant *k*_q_ were obtained using the Stern–Volmer equation to measure the quenching efficiency:

(1) $$F_{0}/F=1+k_{\mathrm{sv}}\left[Q\right]=1+k_{q}\tau_{0}\left[Q\right]$$

where *F*_0_ and *F* represent the fluorescence intensities of the PR‐LBD before and after the titration of bisphenol analogs, respectively. [Q] represents the concentration of bisphenol analogs, and τ_0_ represents the average fluorescence lifetime of protein molecules in the absence of quenching agents and is equal to 10^−8^ s.

To better quantify the degree of BPA interference and that of other bisphenol analogs on the PR‐LBD, the Scatchard equation was applied to calculate the binding constant k_a_ when quenching occurred:

(2) $$\log\left(\left[F0-F\right]/F\right)=\mathrm{logka}+\mathrm{nlog}\left[Q\right]$$

where *n* represents the number of binding sites of bisphenol analogs to the PR‐LBD.

In addition, we quantitatively analyzed the fluorescence enhancement data of bisphenol analogs combined with PR‐LBD according to the following equation developed by Li et al.^[^ ^72^ ^]^ to determine the binding constant *k*_a_:

(3) $$\left(F\infty -F0\right)/\left(\mathrm{Fx}-F0\right)=1+1/\left(\mathrm{ka}\left[Q\right]\right)$$

where *F*_0_, *F*, and *F*_∞_ represent the fluorescence intensity of PR‐LBD when bisphenol analogues are absent, when the intermediate concentration of bisphenol analogues is added, and when their interaction reaches saturation, respectively.

According to the thermodynamic parameters calculated using the van't Hoff equation, the noncovalent binding mode of bisphenol analogs forming complexes with PR‐LBD can be better understood:

(4) $$\ln\left(k2/k1\right)=\Delta H\;\left(1/T1-1/T2\right)/R$$

(5) $$\Delta G=-\mathrm{RTln}k_{a}$$

(6) $$\Delta S=-\left(\Delta G-\Delta H\right)/T$$

where *k*_1_ and *k*_2_ correspond to the binding constants at *T*_1_ and *T*_2_, respectively, Δ*S* represents the change in entropy, Δ*H* represents the change in enthalpy, and Δ*G* represents the change in Gibbs free energy.

### Secondary Structure Examination

As described previously,^[^ ^58^ ^]^ a Chirascan spectrometer (Applied Photophysics Ltd., Leatherhead, Surrey, U.K.) was used to measure the circular dichroism (CD) spectra of PR‐LBD combined with bisphenol analogs at 180–260 nm. Data were scanned three times for each spectrum. Background CD spectra were subtracted from the baseline correction. The measured CD spectral data are expressed in millidegrees (mdeg). Chirascan was used to smoothen the curves, and CDNN was used to analyze the content of each secondary structure.

### Cytotoxicity Test

MCF‐7 cells were maintained in the corresponding special culture medium at 37 °C with 5% CO_2_ and 95% humidity. The cytotoxicity induced by bisphenol analogs was determined using the CCK‐8 assay. To this end, 5 × 10^4^ mL^−1^ MCF‐7 cells were seeded in 96‐well plates. After 24 h pre‐culture, cells were exposed to bisphenol analogs at different doses (10^−11^, 10^−10^, 10^−9^, 10^−8^, 10^−7^, and 10^−6^ m) for 24 h. Then, 10 µL CCK‐8 solution was added to each well, and the 96‐well plate was incubated for an additional 2 h. Using an enzyme labeling instrument (Bio‐Rad), the corresponding absorbance was measured at a wavelength of 450 nm and a proliferation curve was drawn.

### Wound Healing Assay

MCF‐7 cells (7 × 10^5^ cells mL^−1^) were seeded in 24‐well plates containing medium without FBS. A denuded zone of constant width was created by scraping the monolayers with a sterile white micropipette tip (0 h). The monolayers were then washed twice with PBS to remove cellular debris. Subsequently, the cells were exposed to bisphenol analogs for 24 h. The same scratched monolayers were visually captured using an inverted microscope (Olympus, Tokyo, Japan) at 200× magnification. The distance between the cells within the scraped zone was measured from three independent experiments.

### Cell Cycle Detection

MCF‐7 cells were plated in 6‐well culture plates at an initial density of 3×10⁵ cells/well and allowed to adhere overnight under standard culture conditions (37 °C, 5% CO_2_). The adherent cells were subsequently exposed to bisphenol analogues (BPA, BPB, BPE, BPF, BPS, and BPAF) at a concentration of 1×10^−10^ m for 24 h. Following chemical treatment, cells were washed once with phosphate‐buffered saline (PBS) and fixed with 75% ethanol overnight at 4 °C. Before staining, the fixation solution was removed by washing with PBS. Cellular pellets were then treated with 100 µL RNase A solution (1 mg mL^−1^) and incubated at 37 °C for 30 min in a temperature‐controlled water bath. Subsequently, cells were resuspended in 400 µL propidium iodide (PI) staining solution (50 µg mL^−1^) and maintained at 4 °C for 30 min under light‐protected conditions. Cell cycle analysis was performed using an Attune NxT flow cytometer (Thermo Fisher Scientific, USA) with appropriate excitation/emission settings (488 nm/617 nm) for PI detection.

### Transwell Invasion Analysis

Thaw matrigel matrix (BD Biosciences, USA) was thawed on ice overnight. The 50 µL matrigel diluted with cold serum‐free medium (1:10) was added to the upper chamber of a 24‐well Transwell insert (8 µm pore size) and incubated at 37 °C for 4 h to allow polymerization. MCF‐7 cells, after treated with 1 × 10^−10^ m bisphenol analogs (BPA, BPB, BPE, BPF, BPS, and BPAF), were seeded into the upper chamber at a density of 1 × 10^5^ cells/well in serum‐free medium. The lower chamber was filled with complete medium containing 10% fetal bovine serum as a chemoattractant. After treatment, cells on the upper membrane surface were gently removed using cotton swabs. Cells attached to the lower membrane surface were fixed with 4% paraformaldehyde for 20 min, followed by staining with 0.1% crystal violet solution for 15 min. The membranes were washed three times with PBS to remove excess stain and then air‐dried. Cells attached to the lower membrane were visualized under an inverted light microscope (Olympus, Tokyo, Japan) at 100× magnification.

### PR Protein/mRNA Determination

After seeding in 60 mm petri dishes (5 × 10^5^ mL^−1^), MCF‐7 cells were exposed to bisphenol analogs for 24 h. Cells were harvested after washing with PBS, and the protein and mRNA expression levels of the PR were detected.

For protein level, 25 µg protein was electrophoresed for each sample and transferred to a PVDF membrane. The membranes were blocked with PBST containing 5% skim milk powder and Anti‐Progesterone Receptor Rabbit Polyclonal Antibody (Servicebio GB11262, 1:700) at 4 °C overnight. Then, the membrane was washed with PBST three times for 10 min each time. The membranes were then incubated with the secondary antibody (1:3000, Goat Anti‐Rabbit IgG H&L / HRP) for 40 min and washed again with PBST three times for 10 min each time. Imaging was performed using an Integrated Chemiluminescence Imaging Analysis System (Beijing Baygene Biotech Co., Ltd.). Lastly, the analysis was carried out using ImageJ software.

Total RNA was extracted and transcribed to the corresponding complementary DNA (kits were purchased from Promega (Beijing) Biotech Co., Ltd., Beijing, China). A mixture of template cDNA and primer pairs for *PGR*, GoTaq qPCR Master Mix, and RNase‐free H_2_O was prepared for quantitative real‐time PCR (qPCR) using a qTOWER 2.2 Real‐Time PCR system (Analytik Jena AG, Jena, Germany). The expression levels of *PGR* relative to the internal reference gene (glyceraldehyde‐3‐phosphate dehydrogenase, Gapdh) were calculated using the ΔΔCT method.

### Cellular Thermal Shift Assay (CETSA) in Vitro and in Vivo

MCF‐7 cells were seeded in T75 culture flasks (NEST 708003). Once the cell density reached 70%–80%, DMSO was added to the control group, and bisphenol analogs diluted in DMSO were added to the experimental group at a final concentration of 10^−6^ m. After 24 h of treatment with BPs, the cells in each T75 flask were digested with 2 mL of trypsin, then centrifuged and the supernatant was discarded. The cells were then resuspended in 1 mL PBS containing a protease inhibitor (PMSF), centrifuged at 1500 rpm for 5 min, and the supernatant discarded. The cells were resuspended in 480 µL PBS with PMSF and evenly distributed into eight PCR tubes, each containing 100 µL of cell suspension. A gradient denaturation was performed [the temperature was set according to pre‐experiment results: 43.5, 45.0, 46.8, 48.6, 50.4, 52.2, 54.0 °C] for 10 min. The cell suspension was transferred to 1.5 mL Eppendorf tubes.

The cells were then subjected to repeated freeze‐thaw cycles (five times) using liquid nitrogen to lyse them. The lysates were sonicated for 10 min in an ultrasonic cleaner, followed by centrifugation at 15 000 rpm for 20 min at 4 °C. The supernatant was collected and mixed with a 1/4 volume of protein loading buffer and heated at 95 °C for 10 min to denature the proteins. Western blotting was then performed as described in Section 2.9.

Analysis was conducted using ImageJ, and GraphPad Prism (version 9.0; GraphPad Software, Inc.) was used to plot the data and calculate the change in Tm50 (ΔTm50, defined as the difference between the Tm50 of the BPs treatment group and the control group).

For in vivo detection, tumor tissues were collected from tumor‐bearing mice exposed to solvent control (corn oil) and 30 µg kg^−1^ BPAF dissolved in corn oil. Approximately 30 mg of tumor tissue was collected per mouse, and 300 µL of PBS containing PMSF was added on ice, along with grinding steel beads. The tissues were homogenized in a tissue homogenizer until no tissue chunks remained. The samples were then subjected to three freeze‐thaw cycles using liquid nitrogen for cell lysis. The lysates were centrifuged at 12 000 rpm for 15 min at 4 °C to remove cell debris, and the supernatant was collected. The volume was recorded for further analysis.

The protein concentration in the supernatant was quantified using a bicinchoninic acid (BCA) protein assay kit (SW201‐02; SEVEN). The supernatant was diluted with PBS containing PMSF to a concentration of 3 µg µL^−1^ for PR detection. A sample volume of 20 µL was loaded per well, and the protein samples were heated to 46 °C for 10 min. Soluble components were separated by centrifugation at 12 000 rpm for 15 min at 4 °C. After adding a 1/4 volume of protein loading buffer to each tube, the samples were denatured by heating at 95 °C for 10 min. After another centrifugation step, western blotting was performed to measure PR protein levels in the tumor tissue. Anti‐Progesterone Receptor Rabbit Polyclonal Antibody (Servicebio GB11262, 1:800) was used as the primary antibody, and β‐actin (Servicebio ZB15001, 1:2000) was used as the internal control for protein normalization.

### Immunofluorescence Staining

MCF‐7 cells (2 × 10^4^–3 × 10^4^ mL^−1^) were inoculated into a coated culture plate (Costar24‐well Clear) and incubated at 37 °C and 5% CO_2_ for 24 h. After treatment with BPs for 24 h, MCF‐7 cells were fixed with 4% paraformaldehyde and permeabilized with 0.1% Triton X‐100. After washing with PBS, cells were blocked with goat serum and incubated with Anti‐Progesterone receptor Rabbit pAb as the primary antibody at 4 °C overnight. After washing with PBS twice, the cells were incubated with AlexaFluor488‐bound secondary antibody at room temperature for 1 h, and then incubated with the DAPI working solution. Lastly, the plates were sealed with anti‐fluorescence quenchant and the images were obtained using an OLYMPUS imaging fluorescence microscope (Olympus, Tokyo, Japan).

Tumor paraffin tissue sections (*n* = 3) were baked in an oven set at 60 °C for 2 h. The sections were then dewaxed and rehydrated using xylene and graded ethanol (100%, 95%, 75%), followed by washing with distilled water. Subsequently, the sections were incubated in preheated 100 °C sodium citrate buffer (pH = 6) for 15 min. After cooling to room temperature, the sections were permeabilized with 0.2% Triton X‐100 for 45 min. After washing with PBS, the sections were blocked with goat serum at 37 °C for 30 min. Then, the sections were incubated with rabbit polyclonal antibodies against progesterone receptor (1:600) overnight at 4 °C. After washing with PBS three times, Alexa Fluor 488‐conjugated secondary antibody (1:200) was added, and the sections were incubated at room temperature in the dark for 1 h. DAPI was then applied for nuclear staining prior to the sections being mounted with an anti‐fade reagent and imaged using an OLYMPUS fluorescence microscope (Olympus, Tokyo, Japan).

### Data Collection from the CTD and GEO Database

To explore the possible relationship between bisphenol analogs and breast diseases, the CTD was screened using the keyword *BPA* (last accession 10/27/2022). The disease category was filtered with *Breast Cancer*. The core genes of *Breast Neoplasms* were collected and summarized.

Core genes were also collected from a GEO dataset (GSE85350) using the criteria of |log_2_(fold change (FC))| > 1 and *p* < 0.05, which provided alterations in the MCF‐7 transcriptome induced by BPA and four BPA analogs. The core genes included in all the exposure groups were summarized.

Lastly, the intersections of the genes summarized above in the CTD and GEO datasets were filtered.

### ToxPi Analysis

Toxicological Priority Index software (ToxPi_V2.3) was used to rank the toxicity of the BPs.^[^ ^74^ ^]^ Thirteen parameters were divided into five classes (binding affinity of molecular docking and MD was attributed to in silico binding, PR_ΔG_298, PR_αhelix, PR_βsheet, PR_βturn and Random coil was attributed to chemical analysis, ΔTm50 of CESTA results was attributed to in vitro binding, the results of the CCK‐8, wound healing assay and transwell results were attributed to cytotoxicity, PR mRNA and protein expression was attributed to PR expression). Furthermore, the Toxpi values for the binding affinity score and PR_ΔG_298 were converted from negative to positive values, while the values for PR_αhelix, PR_βsheet, PR_βturn, and random coil were replaced with the absolute differences between each experimental group and the control group. The weight was set to 20% for every class, and the data were made uniform before calculating the ToxPi score.

### PR Inhibitor Experiments

MCF‐7 cells were exposed to 0.6 × 10^−9^ m Mifepristone (RU486) for 4 h and then subsequently to either 0.1% DMSO or 10^−10^ M BPAF for 24 h. Then, protein expression of PR, wound healing assay, and transwell experiment were conducted as mentioned above.

### Animal Model

Female BALB/c nude mice (5 weeks old) were acclimatized for 1 week under in a climate‐controlled room (temperature: 22 ± 2 °C; humidity: 40 ± 5%; and photoperiod: 12 h of light/12 h of dark), with free access to water and food. MCF7‐Luc cells (ATCC‐derived, stably transfected with luciferase by Shanghai Dinuoxinan Bio‐Technology Co., Ltd.) were cultured in DMEM supplemented with 10% FBS and 1% penicillin/streptomycin. The cells were harvested and resuspended in PBS at a density of 5 × 10⁸ cells mL^−1^ for inoculation. The mice were anesthetized with 240 mg kg^−1^ tribromoethanol (Avertin) via intraperitoneal injection. A 100 µL cell suspension (5 × 10⁷ cells/mouse) was orthotopically injected into the right mammary fat pad. Post‐inoculation, mice were placed on a warming pad at 37 °C until fully recovered and maintained in individually ventilated cages. At 14 d post‐inoculation, mice bearing tumors (≈100 mm^3^) were randomized into two groups: (1) Control (corn oil vehicle), (2) BPAF (30 µg kg^−1^ b.w.). Treatments were administered daily by oral gavage (200 µL/mouse) for 14 d. Body weight and tumor volume (calculated as *V* = *L* × *W* ^2^/2, where *L* = length and *W* = width) were measured every 2 d. Bioluminescence imaging (IVIS Spectrum, PerkinElmer) was performed weekly to monitor tumor progression. After exposure for 14 d, mice were euthanized at the study endpoint, and tumors were excised, weighed, and divided for formalin fixation or snap‐freezing in liquid nitrogen and stored at −80 °C for downstream analyses. All experimental protocols were approved by the Committee of Scientific Research at Shanxi University.

### Statistical Analysis

Data are presented as mean ± standard deviation (SD). For multiple comparisons, one‐way ANOVA with Dunnett's correction was applied when equal SDs were assumed. In cases where the assumption of equal SDs was violated, Brown–Forsythe and Welch ANOVA tests with Dunnett's T3 correction were used. For pairwise comparisons, an unpaired two‐tailed *t*‐test without correction was employed when both populations exhibited equal SDs. If the SDs were unequal, an unpaired two‐tailed *t*‐test with Welch's correction was applied. A *p*‐value of less than 0.05 was considered statistically significant. All statistical analyses were performed using GraphPad Prism.

## Conflict of Interest

The authors declare no conflict of interest.

## Supporting information



Supporting Information

## Data Availability

The data that support the findings of this study are available from the corresponding author upon reasonable request.
