# Supplementary material for: Replacing BPA: Structural Substitutes BPAF Binding to the Progesterone Receptor Elevates Breast Cancer Risk
Source: Adv Sci (Weinh). 2025 Nov 7;13(4):e02444. doi: 10.1002/advs.202502444 (PMC12822391; doi:10.1002/advs.202502444)
Supplement: Supplementary file 1 — Supporting Information [file ADVS-13-e02444-s001.docx]

Supplement information

***Replacing BPA: Structural Substitutes BPAF Binding to the Progesterone Receptor Elevates Breast Cancer Risk***

Xiaotong Ji^1,^ *^, #^, Peilin Li^1, #^, Haoyang Wu^1^, Linzhuo Shen^1^, Xiaoyun Wu^2^, Peiyun Jiang^1^, Yating Li^1^, Xiaozheng Zhang^3^, Huifeng Yue^2,^ *

1 Department of Environmental Health, School of Public Health, Shanxi Key Laboratory of Environmental Health Impairment and Prevention, MOE Key Laboratory of Coal Environmental Pathogenicity and Prevention, Shanxi Medical University, Taiyuan, Shanxi 030001, PR China

2 College of Environment and Resource, Research Center of Environment and Health, Shanxi Key Laboratory of Coal-based Emerging Pollutant Identification and Risk Control, Shanxi University, Taiyuan, Shanxi 030006, PR China

3 Department of Biochemistry and Molecular Biology, Shanxi Key Laboratory of Birth Defect and Cell Regeneration, Shanxi Medical University, Taiyuan, Shanxi, 030001, PR China

Conflict of Interest

All authors declare that they have no conflicts of interest.

# These authors contributed equally to this work.

* Corresponding author. Tel./ Fax: +86-351-7010600

E-mail: jixiaotong@sxmu.edu.cn, yuehuifeng@sxu.edu.cn

**Cover Sheet**

| Manuscript Title | Replacing BPA: Structural Binding to the Progesterone Receptor by substitutes BPAF Elevates Breast Cancer Risk |
| --- | --- |
| Complete List of Authors | Xiaotong Ji^1,^ *^, #^, Peilin Li^1, #^, Haoyang Wu^1^, Linzhuo Shen^1^, Xiaoyun Wu^2^, Peiyun Jiang^1^, Yating Li^1^, Xiaozheng Zhang^3^, Huifeng Yue^2,^ * |
| Total Pages | 5 |
| Total Texts | 0 |
| Total Tables | 1 |
| Total Figures | 2 |

**Table S1.** Tm50 Values from nonlin fit of melting curves for PR treated with different BPs.

|  | DMSO | BPE | BPAF | BPF | BPA | BPB | BPS |
| --- | --- | --- | --- | --- | --- | --- | --- |
| Tm50 (℃) | 46.82 | 48.12 | 48.71 | 47.07 | 48.79 | 48.14 | 47.21 |


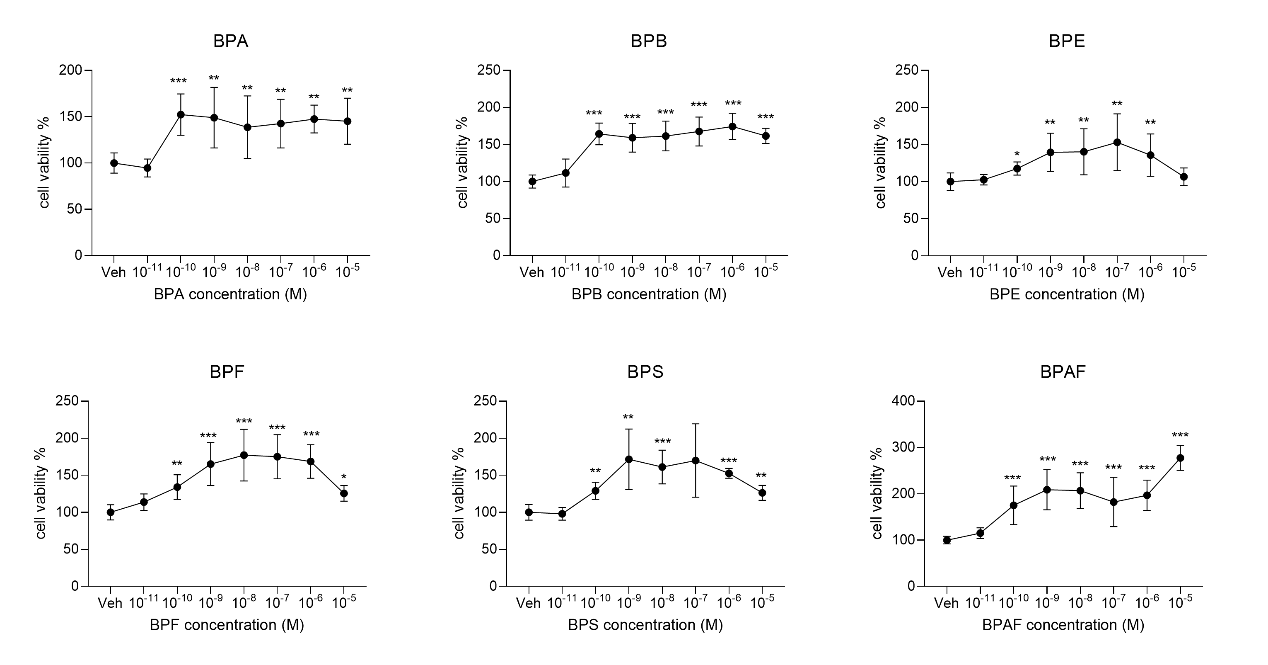


**Figure S1.** Proliferative analysis (n = 4) of BPs exposure on MCF-7.


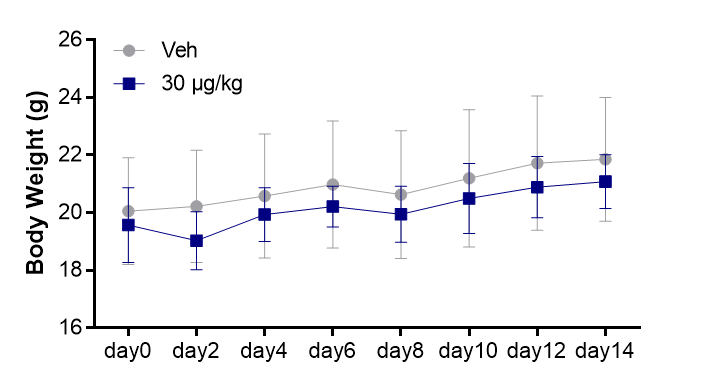


**Figure S2**. Body weight of mice of xenograft models.
